# Spontaneous Recovery From Post-hemorrhagic Obstructive Hydrocephalus: A Rare Case in an Elderly Patient

**DOI:** 10.7759/cureus.99888

**Published:** 2025-12-22

**Authors:** Eduardo Trejo Olguin, Saul R Amparan, Arturo M Rodriguez Saldivar, Leopoldo Pérez García, Christian Félix Montiel, Mariana Mercado Flores, Jesus Alberto Morales Gomez, Angel Martínez Ponce De Leon

**Affiliations:** 1 Neurosurgery, Hospital Universitario Dr. José Eleuterio González, Monterrey, MEX; 2 Neuroradiology, Hospital Universitario Dr. José Eleuterio González, Monterrey, MEX; 3 Neurosurgery , Universidad Autónoma de Nuevo León, Nuevo León, MEX; 4 Neurological Surgery, Hospital Universitario Dr. José Eleuterio González, Monterrey, MEX

**Keywords:** elderly patient, hydrocephalus, post-hemorrhagic hydrocephalus, post-obstructive hydrocephalus, spontaneous resolution

## Abstract

A 70-year-old woman with type 2 diabetes mellitus presented with a sudden, severe headache and altered mental status. Non-contrast cranial CT performed at an external facility revealed hydrocephalus secondary to spontaneous intraventricular hemorrhage localized to the aqueduct of Sylvius. Upon admission to our institution, follow-up neuroimaging demonstrated complete spontaneous resolution of the hydrocephalus, without evidence of aqueductal obstruction and without requiring neurosurgical intervention. This case represents an uncommon instance of spontaneous resolution of obstructive hydrocephalus secondary to intraventricular hemorrhage in an elderly patient, underscoring the importance of individualized assessment and close neurological monitoring before undertaking invasive procedures.

## Introduction

Intracerebral hemorrhage (ICH) is one of the most severe cerebrovascular events, and primary intraventricular hemorrhage (PIVH) represents only 1.9%-3.3% of all cases [1]. In adults, common causes include hypertension, arteriovenous malformations, aneurysms, intraventricular tumors, and coagulopathies, while rare conditions such as Moyamoya disease, chronic hypertension, and diabetes mellitus may increase venous fragility and predispose to spontaneous bleeding [2].

A critical complication of PIVH is acute obstructive hydrocephalus, resulting from cerebrospinal fluid (CSF) flow blockage by intraventricular clots, particularly within the aqueduct of Sylvius. This can cause intracranial hypertension and neurological deterioration, typically requiring surgical drainage. However, rare reports describe spontaneous resolution of hydrocephalus, attributed to intrinsic CSF fibrinolytic activity and gradual restoration of ventricular patency [3,4].

## Case presentation

**Figure 1:**
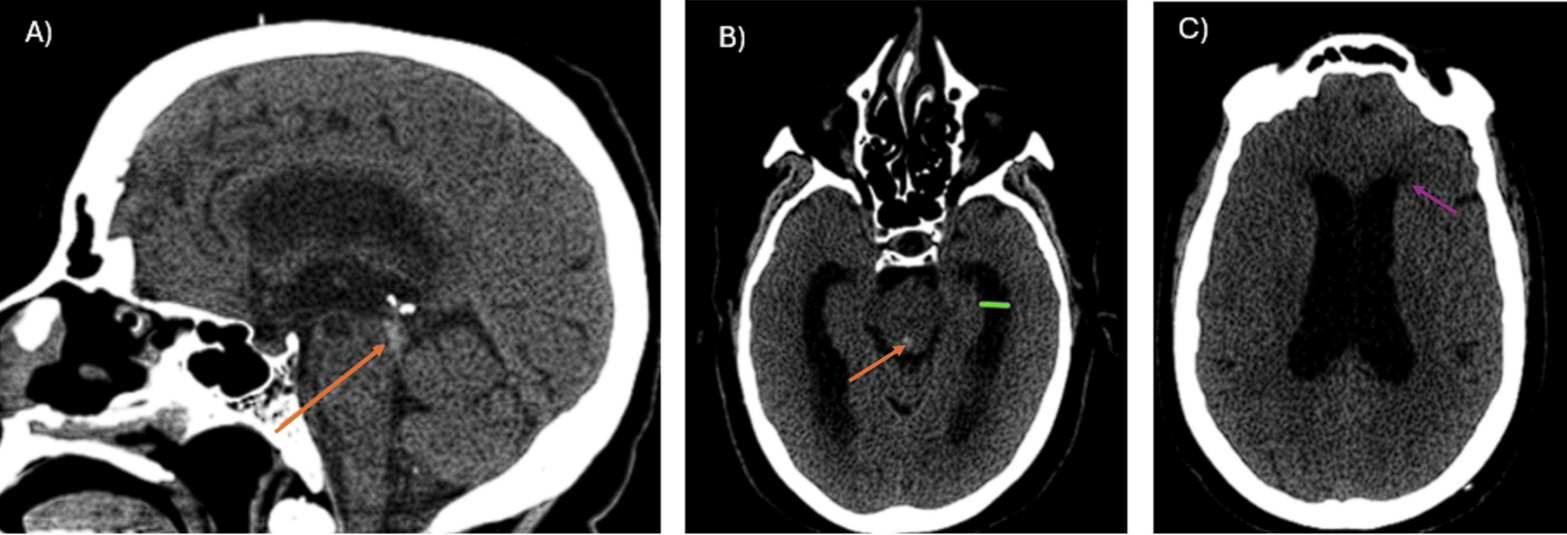
Non-contrast CT images. (A) Sagittal non-contrast CT image obtained at the onset of clinical symptoms, demonstrating a clot (orange arrow) with a density of 60 HU, located within the mesencephalic aqueduct. (B, C) Axial CT images obtained at the onset of clinical symptoms, showing enlargement of the bodies of both lateral ventricles and temporal horns (green line), as well as transependymal edema (purple arrow). A clot is identified within the mesencephalic aqueduct (orange arrow), representing direct and indirect signs of obstructive hydrocephalus.

A 70-year-old woman with a 10-year history of type 2 diabetes mellitus, controlled with metformin/glipizide, presented with a sudden, severe headache, confusion, lethargy, and incoherent speech, with a Glasgow Coma Scale (GCS) score of 12/15. At a local clinic, her blood pressure was 190/100 mmHg, with normal glucose levels and no focal neurological deficits. A CT of the head revealed a spontaneous periventricular hemorrhage involving the aqueduct of Sylvius, producing acute obstructive hydrocephalus (Evans index > 0.30) (Figure 1).

**Figure 2:**
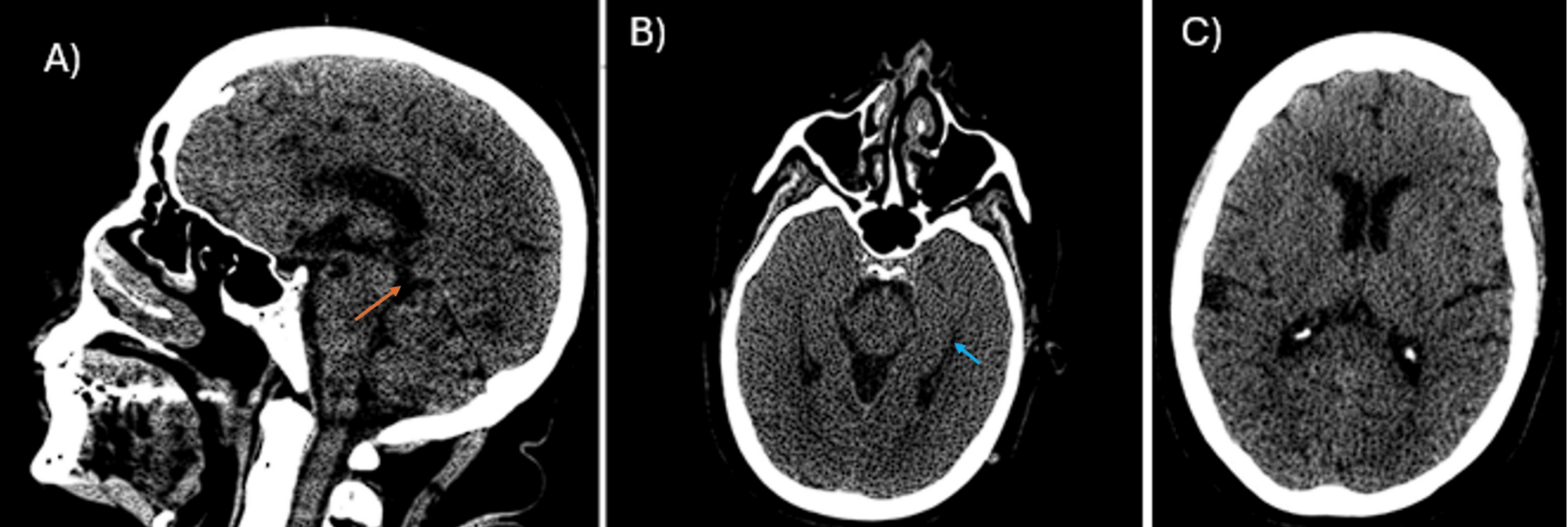
Non-contrast CT images. (A, B) Sagittal and axial non-contrast CT images acquired after the spontaneous resolution of the patient’s symptoms, showing complete resolution of the clot located within the mesencephalic aqueduct (orange arrow) and normalization of the size of the temporal horns of the lateral ventricles (blue arrow). (C) Axial non-contrast CT image acquired after the spontaneous resolution of symptoms, showing absence of transependymal edema and normal diameters of the bodies of the lateral ventricles.

Upon transfer to a tertiary center three days later, she was neurologically intact (GCS score of 15). Follow-up CT showed complete resolution of the hemorrhage and normalization of ventricular size (Figure 2).

**Figure 3:**
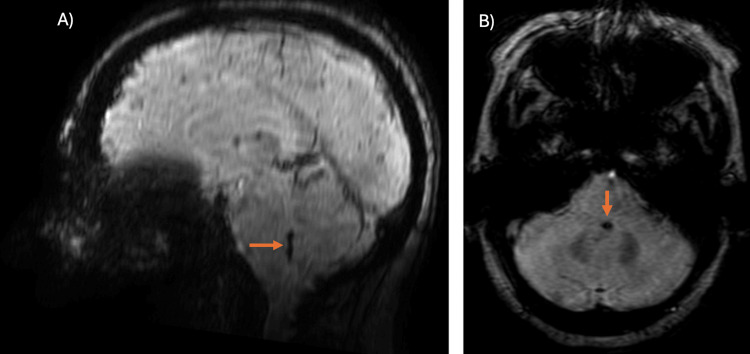
Susceptibility-weighted angiography sequence MRI. (A, B) Sagittal and axial susceptibility-weighted angiography sequence showing hemosiderin remnants in the median sulcus and obex of the fourth ventricle (orange arrows).

**Figure 4:**
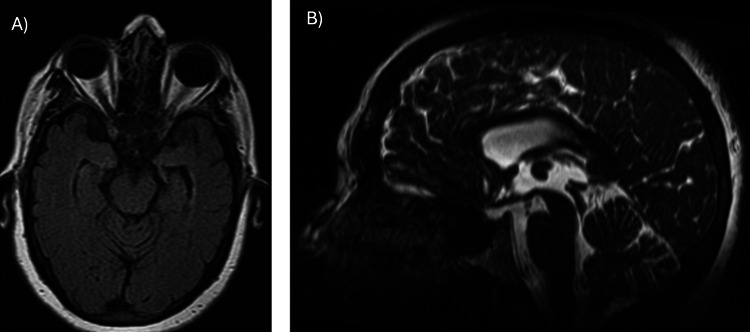
Fluid-attenuated inversion recovery and fast imaging employing steady-state acquisition sequence image. (A, B) Axial fluid-attenuated inversion recovery and sagittal fast imaging employing steady-state acquisition sequence images showing resolution of the enlargement of the temporal recesses of the lateral ventricles and absence of thrombus in the mesencephalic aqueduct.

**Figure 5:**
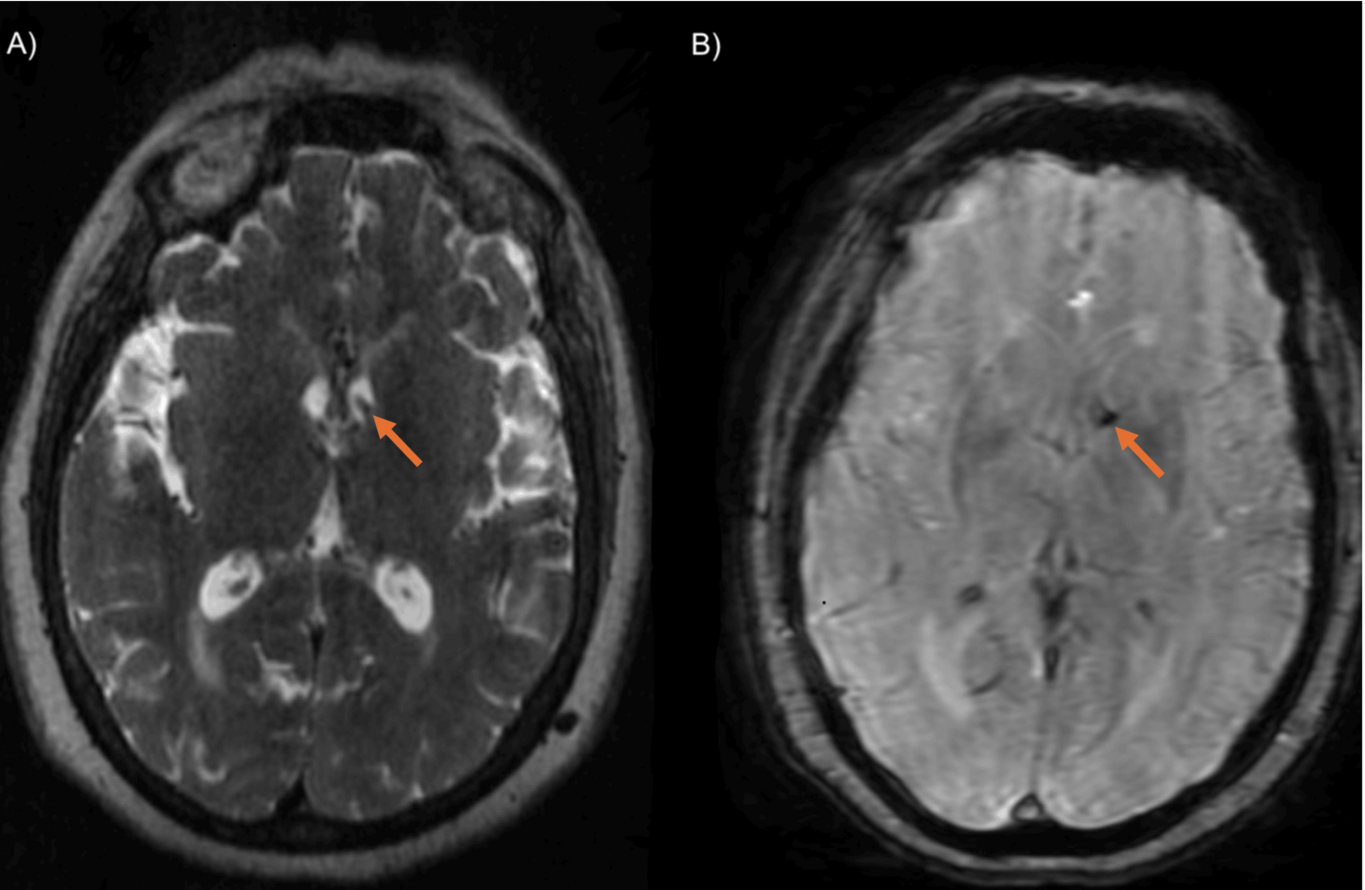
Fast imaging employing steady-state acquisition and susceptibility-weighted angiography sequences. Axial images in fast imaging employing steady-state acquisition and susceptibility-weighted angiography sequences showing an area suggestive of the hemorrhage origin, likely resulting from rupture of an ependymal vein (orange arrows).

MRI excluded secondary causes, showing only residual hemosiderin deposits consistent with a venous origin (Figures 3-5). The patient remained clinically stable and was discharged asymptomatic after 72 hours without neurosurgical intervention.

At the two-month follow-up, the patient’s GCS score was 15, her pupils were isocoric (3 mm) and normoreflective, and her muscle strength was rated 5/5 on the Lovett scale in all four limbs. She had no sensory or vestibulocerebellar abnormalities. The patient was scheduled for a follow-up appointment in two months.

## Discussion

PIVH in adults is rare, accounting for fewer than 2% of all spontaneous ICHs. Chronic hypertension remains the predominant etiological factor, and, in this case, an acute hypertensive episode likely precipitated the spontaneous bleeding localized to the aqueduct of Sylvius [1,5,6].

Pathophysiologically, intraventricular blood can transiently obstruct CSF flow, particularly at the aqueduct, a narrow and functionally critical conduit. Such obstruction typically leads to life-threatening acute hydrocephalus that often necessitates urgent surgical decompression. In exceptional cases, however, spontaneous resolution occurs, a phenomenon described as transient obstructive hydrocephalus [3,4].

Comparative imaging in this case revealed complete resolution of both hemorrhage and hydrocephalus within 72 hours, consistent with a self-limiting process. Several mechanisms have been proposed to explain spontaneous resolution, including endogenous CSF fibrinolysis, mediated by plasminogen and tissue plasminogen activator, facilitating gradual intraventricular clot dissolution; clot migration or redistribution, driven by cerebral pulsations and respiratory movements, shifting the clot into larger ventricular spaces, thereby alleviating obstruction; pressure-gradient reabsorption, where dynamic intracranial pressure variations promote clot fragmentation and clearance; combined CSF dynamics and mild inflammatory response, where transient inflammatory changes alter CSF viscosity and assist in the removal of blood products; and limited hemorrhagic volume with partial aqueductal patency, permitting compensatory CSF flow and preventing severe intracranial hypertension [1-4].

Spontaneous resolution of obstructive hydrocephalus remains rare but clinically significant, as it may preclude unnecessary neurosurgical intervention in stable patients. In this case, radiological evidence supported the hypothesis of clot redistribution and gradual aqueductal recanalization.

An additional mechanism to consider in the pathophysiology of intraventricular hemorrhage is spontaneous rupture of subependymal or terminal intraventricular veins. Classic neuropathological studies demonstrated that abrupt increases in venous pressure can rupture fragile periventricular or choroidal veins, particularly in contexts of venous congestion, hypoxia, or acidosis. Such venous ruptures may cause intraventricular bleeding even in the absence of arterial injury [7,8]. This mechanism may explain cases such as ours, where no parenchymal focus or arterial malformation was identified, but imaging suggested a venous origin of the hemorrhage.

Taken together, this case contributes to the understanding of the natural history of post-hemorrhagic hydrocephalus, illustrating that spontaneous aqueductal recanalization and CSF flow restoration can occur through intrinsic physiological mechanisms. Meticulous monitoring and patient-specific decision-making are critical when opting for conservative management in similar clinical settings.

## Conclusions

In neurologically stable patients without evidence of progressive intracranial hypertension, close observation with CT or MRI before and after the resolution and a proper follow-up during the first year post-resolution might represent a safe and effective alternative to invasive procedures. The spontaneous resolution observed in this case likely resulted from partial clot lysis and migration, with subsequent restoration of ventricular patency. The hemorrhage likely originated from rupture of fragile subependymal veins, favored by chronic hypertension and diabetes-related microvascular fragility. This case expands current knowledge on the natural course of post-hemorrhagic hydrocephalus and supports the notion that spontaneous autolytic and recanalization mechanisms of CSF circulation may occur in selected patients, warranting further investigation into the physiological and molecular processes underlying this phenomenon.

## References

[1] Weinstein R, Ess K, Sirdar B, Song S, Cutting S (2017). Primary intraventricular hemorrhage: clinical characteristics and outcomes. J Stroke Cerebrovasc Dis.

[2] Hesami O, Kasmaei HD, Matini F, Assarzadegan F, Mansouri B, Jabbehdari S (2015). Relationship between intracerebral hemorrhage and diabetes mellitus: a case-control study. J Clin Diagn Res.

[3] Lusis EA, Vellimana AK, Ray WZ, Chicoine MR, Jost SC (2013). Transient obstructive hydrocephalus due to intraventricular hemorrhage: a case report and review of literature. J Clin Neurol.

[4] Hou K, Zhu X, Sun Y, Gao X, Zhao J, Zhang Y, Li G (2017). Transient acute hydrocephalus after spontaneous intracranial bleeding in adults. World Neurosurg.

[5] Brott T, Thalinger K, Hertzberg V (1986). Hypertension as a risk factor for spontaneous intracerebral hemorrhage. Stroke.

[6] Bako AT, Pan A, Potter T (2022). Contemporary trends in the nationwide incidence of primary intracerebral hemorrhage. Stroke.

[7] Garcia JH, Ho KL (1992). Pathology of hypertensive arteriopathy. Neurosurg Clin N Am.

[8] Martins AN, Kobrine AI, Larsen DF (1974). Pressure in the sagittal sinus during intracranial hypertension in man. J Neurosurg.

